# Identification of Hub Genes Related to the Recovery Phase of Irradiation Injury by Microarray and Integrated Gene Network Analysis

**DOI:** 10.1371/journal.pone.0024680

**Published:** 2011-09-13

**Authors:** Jing Zhang, Yue Yang, Yin Wang, Jinyuan Zhang, Zejian Wang, Ming Yin, Xudong Shen

**Affiliations:** 1 School of Pharmacy, Shanghai Jiao Tong University, Shanghai, China; 2 Department of Internal Medicine, No. 455 Hospital, Shanghai, China; Centro de Investigación Príncipe Felipe, Spain

## Abstract

**Background:**

Irradiation commonly causes long-term bone marrow injury charactertized by defective HSC self-renewal and a decrease in HSC reserve. However, the effect of high-dose IR on global gene expression during bone marrow recovery remains unknown.

**Methodology:**

Microarray analysis was used to identify differentially expressed genes that are likely to be critical for bone marrow recovery. Multiple bioinformatics analyses were conducted to identify key hub genes, pathways and biological processes.

**Principal Findings:**

1) We identified 1302 differentially expressed genes in murine bone marrow at 3, 7, 11 and 21 days after irradiation. Eleven of these genes are known to be HSC self-renewal associated genes, including *Adipoq*, *Ccl3*, *Ccnd1*, *Ccnd2*, *Cdkn1a*, *Cxcl12*, *Junb*, *Pten*, *Tal1*, *Thy1* and *Tnf*; 2) These 1302 differentially expressed genes function in multiple biological processes of immunity, including hematopoiesis and response to stimuli, and cellular processes including cell proliferation, differentiation, adhesion and signaling; 3) Dynamic Gene Network analysis identified a subgroup of 25 core genes that participate in immune response, regulation of transcription and nucleosome assembly; 4) A comparison of our data with known irradiation-related genes extracted from literature showed 42 genes that matched the results of our microarray analysis, thus demonstrated consistency between studies; 5) Protein-protein interaction network and pathway analyses indicated several essential protein-protein interactions and signaling pathways, including focal adhesion and several immune-related signaling pathways.

**Conclusions:**

Comparisons to other gene array datasets indicate that global gene expression profiles of irradiation damaged bone marrow show significant differences between injury and recovery phases. Our data suggest that immune response (including hematopoiesis) can be considered as a critical biological process in bone marrow recovery. Several critical hub genes that are key members of significant pathways or gene networks were identified by our comprehensive analysis.

## Introduction

Ionizing radiation (IR) is ubiquitous in our environment and has many beneficial medical and industrial applications. However, IR also carries potential hazards for human beings, particularly for bone marrow (BM) with the ability of rapid self-renewal [1]. In addition, radiotherapy, as a commonly used treatment for various tumors, induces severe toxic effects on normal healthy tissues due to its non-tumor specificity [2]. Due to the significant undesirable side-effects, it would be of importance to study the cellular response to IR at the genetic level.

Acute myelosuppression is highlighted as a common side effect of radiation damage, which mainly damages the rapidly proliferating hematopoietic stem cells (HSCs), hematopoietic progenitor cells (HPCs) and their more mature progeny [3]. The majority of patients recover rapidly from acute symptoms with seemingly complete recovery of peripheral-blood cell counts and BM cellularity. The recovery of BM hematopoietic function can be promoted by combined use of various hematopoietic growth factors (HGFs) [4], [5]. However, large numbers of patients still suffer from defective HSC self-renewal and decreased HSC reserves during long-term BM injury [4]. Current medical management of irradiation patients is not satisfactory. Furthermore, the identification of possible therapeutics to overcome this problem is confounded by the lack of knowledge concerning the IR-induced molecular and genetic pathophysiology [6].

Microarray is an effective approach to monitor global alterations of gene expression and identify genes that are important to IR-induced disease processes [7]. Dai *et al*. identified 34 up-regulated and 69 down-regulated genes in murine bone marrow 6 h after 6.5 Gy gamma radiation when compared to the expression levels in untreated bone marrow. These genes participate in DNA replication/repair, proliferation/apoptosis, cell cycle control and RNA processing [8]. Previous groups have used microarray to determine the effect of acute high-dose or prolonged low-dose IR exposure on gene expression in various tissues, including: kidneys, testes and brain [9]–[13]. The analysis of Gene Ontology and pathway enrichment of these differentially expressed genes have provided basic insight into the molecular pathogenesis of radiation injury and gene regulation induced by IR.

However, these experiments focused more on the injury phase instead of the recovery phase. Global gene expression analyses at the stage of tissue recovery after high-dose IR have not been reported thus far. There are several important pathological processes during the recovery phase which prompt us to study differential gene expression profiles and their interactive relationships. First, surviving victims exposed to a range of 7–10 Gy total body irradiation (TBI) frequently develop a delayed complex hematologic pathology [4], [14]. Additionally, myelosuppression appears during the first days after IR, mainly attributed to the injury of HSCs and mesenchymal stem cells (MSCs). These two stem cells are critical for BM regeneration and hematopoiesis recovery, which is expected to last over an extended time period [3], [15]–[17]. Furthermore, a set of genes involved in hematopoietic recovery, such as, *Cdkn1α*, and *Il12a*, have been reported to be induced at 24 h, or longer, after exposure. These genes are expected to be informative biomarkers and potentially have use as therapeutic targets [18], [19].

In this paper, our hypotheses were: 1) the gene expression profiles of irradiation damaged bone marrow are not identical in injury phase and recovery phase; and 2) hub genes potentially vital to BM recovery can be identified by means of microarray analysis combined with bioinformatics analysis of genetic networks. To prove our hypotheses, we used microarray analysis to monitor differential gene expression in bone marrow at 3, 7, 11 and 21 days after total body irradiation (TBI) compared to sham-irradiated control. Comprehensive bioinformatics analyses were used to enrich datasets for Gene Ontology and pathway information to provide deeper insight into the biological mechanisms at the recovery phase after irradiation injury. Using this approach we were able to predict hub genes that are most likely associated with BM recovery after acute high-dose irradiation and identify molecules that could serve as novel therapeutic targets.

## Results

### Mouse model of irradiation injury

**Figure 1:**
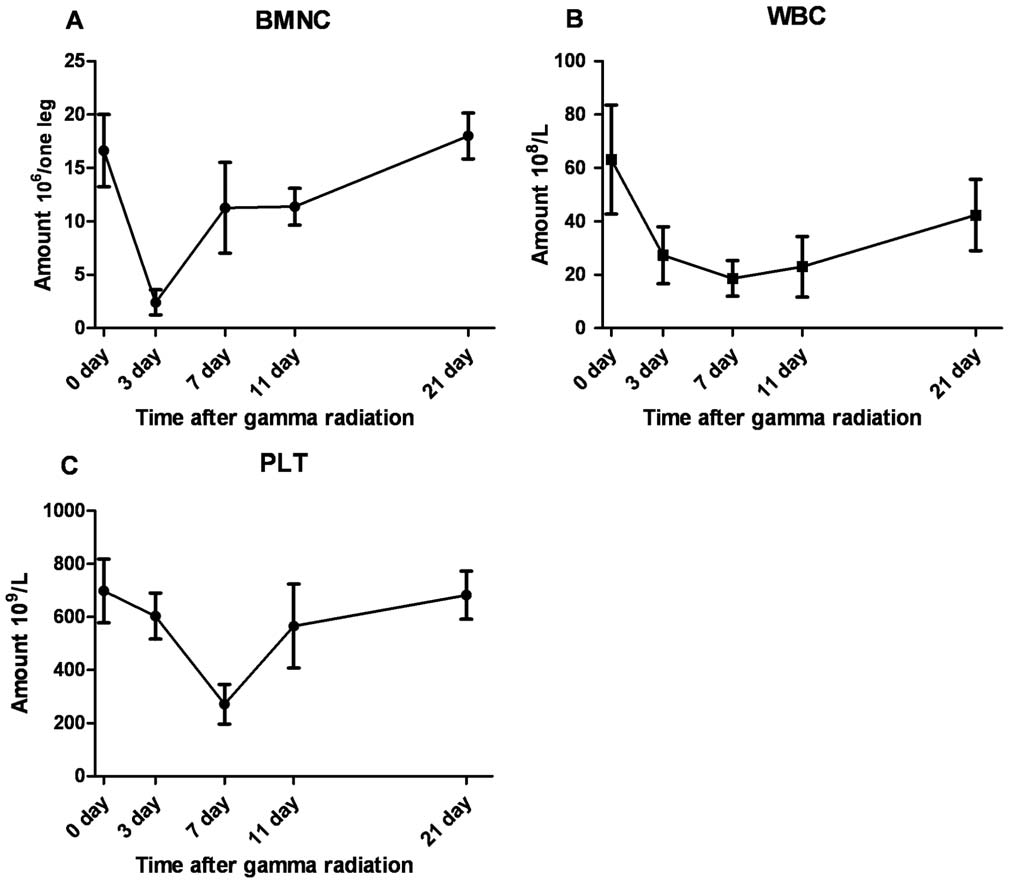
Myelosuppression in mice after ionizing radiation. (A) BMNC counts in mice after IR. (B) WBC counts in mice after IR. (C) PLT counts in mice after IR. We used 6∼8 mice at each time point.

Bone marrow injury and recovery induced by IR were assessed according to peripheral blood cell counts and total bone marrow nucleated cell (BMNC) counts (Figure 1). BMNC counts reached nadir at 3 days after IR (3 DAI) and the number returned to the normal level by 21 DAI (Figure 1A). In addition, peripheral blood cell counts also reduced significantly, including white blood cell (WBC) counts (Figure 1B) and platelet (PLT) counts (Figure 1C). It is note worthy that the number of BMNCs begins to increase at approximate 3 DAI and the recovery of WBCs and PLTs exhibit little delay. The results suggest that damaged bone marrow and hematopoietic function begin to recovery at approximately 3 DAI.

### Microarray data

To investigate gene expression profiles that are associated with BM recovery, mRNA expression in total bone marrow cells was profiled using gene chip at 3, 7, 11 and 21 DAI compared to 0 DAI (sham-irradiation) as control. A total of 1302 genes were identified that showed statistically significant differential expression in irradiated mice at least at one time point when compared to control (*p*-value<0.05) [Table S1]. A subset of these genes is known to have functions that are directly related to HSC self-renewal. By comparing our results with HSC self-renewal and proliferation associated genes reported by Kirouac *et al*. [20], we identified eleven differentially expressed genes in common, *ADIPOQ*, *CCL3*, *CCND1*, *CCND2*, *CDKN1A*, *CXCL12*, *JUNB*, *PTEN*, *TAL1*, *THY1* and *TNF*. Our data show that *Adipoq* and *Cxcl12* were up-regulated, which is known to increase HSC proliferation; conversely, *Ccl3* and *Tnf* were down-regulated, which is known to repress HSC proliferation [20]. Based on these data, cell-extrinsic and cell-intrinsic regulation networks can be constructed, which are expected to be comprised of genes that regulate damaged bone marrow regeneration and have a central role in the control of HSC proliferation.

### Cluster analysis of significant differential genes

**Figure 2:**
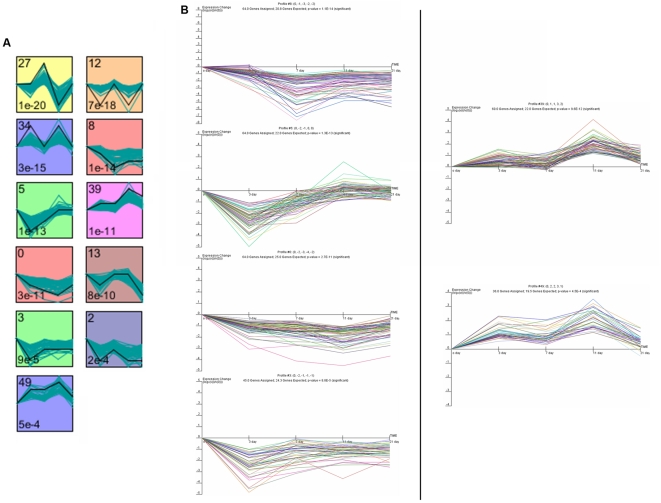
STEM clustering analysis of 1302 significant differential genes in BM after IR. (A) Eleven profiles (*p*<0.001) with a statistically significant number of genes assigned. The number in the top left corner represents the ID of the profile; the bottom left corner shows *p*-value and the green curve shows individual gene expression profiles. (B) Six model profiles detailed in expression graphs. The above images are considered to be the potential main expression profiles in our experiment, with the tendencies consistent with or opposite to that of a myelosuppression change curve (Figure 1). Details of the genes that mapped to each temporal profile are found in Table S2.

The temporal expression pattern of significant differential gene expression was examined by using STEM software, each profile contains a cluster of multiple genes which have similar expression patterns after IR [21]. Eleven significant clusters containing a total of 686 genes were identified (Figure 2, Table S2). Six of these clusters are comprised of genes that were repressed at early time points and then gradually elevated expression levels at later time points, for example profiles 8, 5, 0 and 3; while genes in profiles 39 and 49 had opposite effects. They are the predominant expression profiles in our experiment (Figure 2), and consist of genes with the tendencies consistent with or opposite to that occurred during myelosuppression (Figure 1).

Gene Ontology (GO) analysis was conducted for each pattern based on the EBI database. As shown in Table 1, the high-enrichment GO terms included: metabolic process, response to stimuli, immune response (including hematopoiesis) and cellular component organization. Detailed functional category information for each temporal expression cluster is shown in Table S3.

**Table 1:** The summary of critical functional categories in significant expression patterns.

Function Category	Profile 27	Profile 12	Profile 8	Profile 5	Profile 39	Profile 0	Profile 13
matabolic process	**√**	**√**	**√**	**√**			
response to stimuli	**√**		**√**		**√**		
immune response*	**√**		**√**	**√**	**√**	**√**	**√**
gene expression			**√**				
homeostatic process							**√**
cellular component organization			**√**			**√**	

*Immune response includes the biological process of hematopoiesis.

### GO analysis based on cluster analysis

Further comprehensive GO analysis was applied to differentially expressed genes to gain deeper insight into the main cellular functions that are altered during IR recovery. This was performed according to Gene Ontology enrichment resources available on PubMed [22]. A total of 686 genes from cluster analysis were assigned GO terms (Table S4,S5,S6) [23]. After filtering using the significant criterion of *p*<0.05, we selected 265 genes that fell in the range of key functional classifications. A Comparison of the comprehensive GO analysis and cluster GO analysis, suggests that immune response (including hematopoiesis), cellular component organization, response to stimuli, cellular processes (cell proliferation and adhesion) and signaling processes are the most prominent cellular functions during recovery.

**Figure 3:**
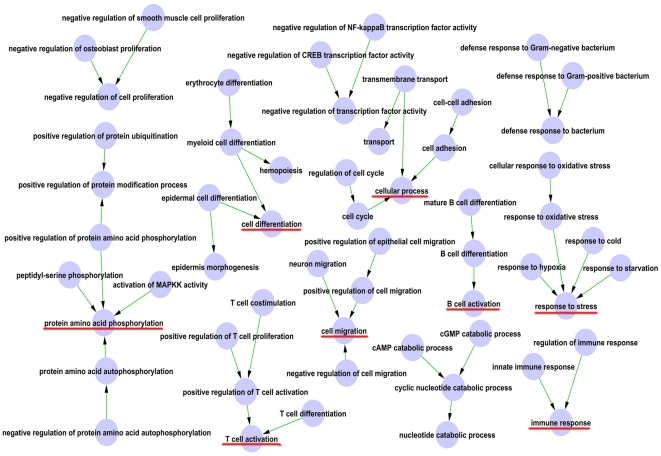
The interaction network of significant GO terms. The node represents one GO term, and the edge between two nodes represents the subordinate relationship between GO terms, with arrow from the lower ranking GO term pointing towards to the higher one.

An interaction network of significant GO terms was assembled into a GO map to depict the relationship among prominent functional categories (Figure 3). Based on the GO map, we can directly and systematically find the subordinate relationship between GO terms (Figure 3 and Table S7). There are 12 subnetworks clustered into four functions: 1) immune (including hematopoiesis): T/B cell activation and immune response; 2) response to stimuli: response to stress and defense response to bacterium; 3) cellular process: cell differentiation, proliferation, migration, adhesion and cycle; 4) cell signaling: protein amino acid phosphorylation, negative regulation of transcription factor activity and cyclic nucleotide catabolic process.

### Dynamic Gene network analysis based on GO analysis

**Figure 4:**
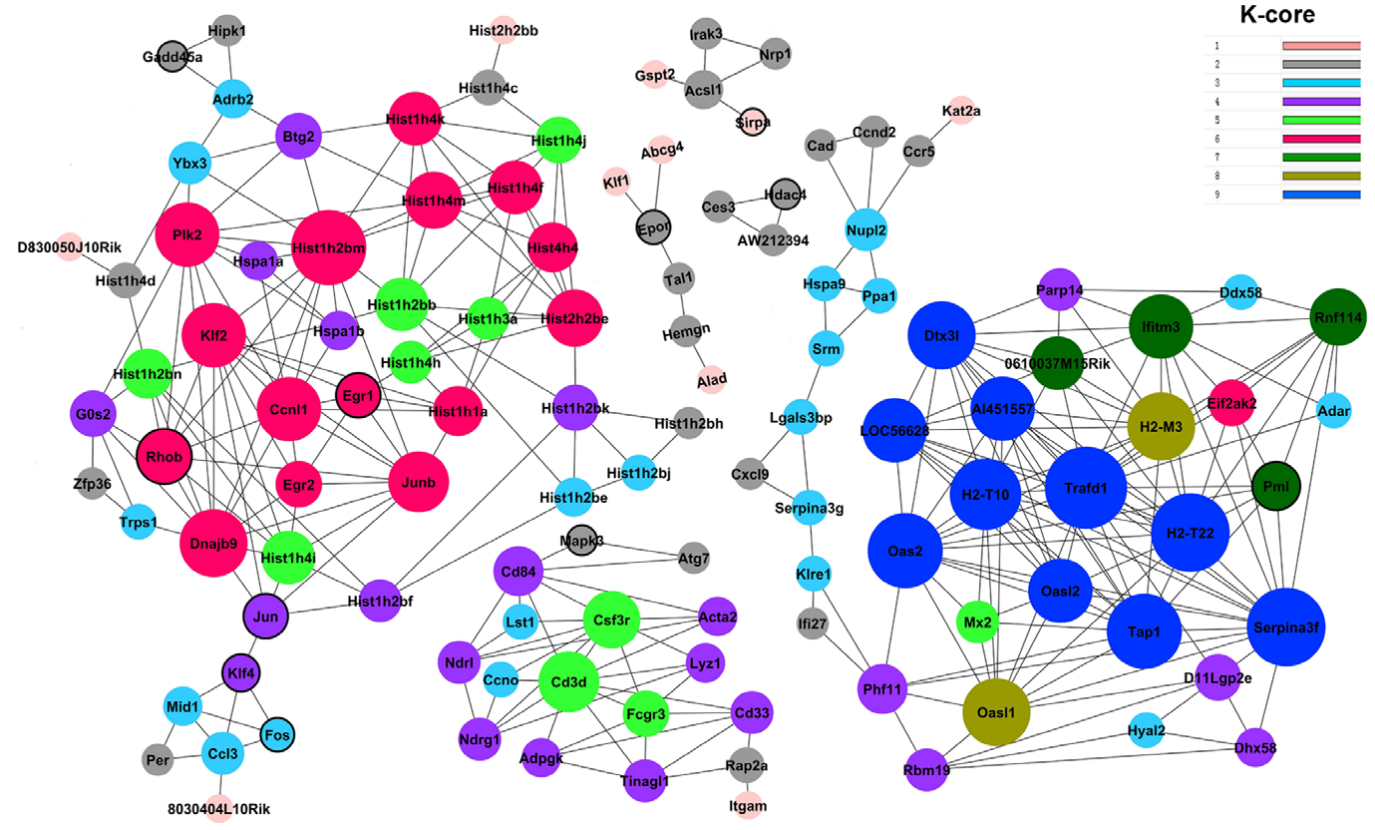
Gene co-expression network. Genes contained in significant GO terms were analyzed and identified by gene co-expression network with the k-core algorithm. Nodes represent the gene (black-cycled node represents gene also appeared in the text-mined gene list); edges indicate the interaction between genes. All the nodes were marked with k-core values. Genes with higher k-core values are more centralized in the network and have a stronger capacity of modulating adjacent genes.

To further narrow down the gene data set for genes that regulate bone marrow recovery, 265 genes selected in GO analysis were analyzed by gene co-expression network with k-core algorithm [24]. Gene co-expression networks were built according to the normalized signal intensity of differentially expressed genes. The network shown in Figure 4 reflects the correlations between genes. Each node describes a given gene, and the relationship between a pair of genes is represented with a line segment. Within the gene network, degree describes the number of links one gene has to others, with the most central the genes in the network having the highest degree values. Clustering coefficient was applied to evaluate the property of a node in network, which represents the connectivity of the adjacent genes with the node. The higher the clustering coefficient, the more complex the interactions of one gene among its neighboring genes [25].

K-core in graph theory was applied to describe the characteristics of the network. A k-core of gene co-expression network usually contains cohesive groups of genes [25]. There are three main k-core subnetworks in our results, including 156 genes (shown in Table S8). The genes with the highest k-core level in each subnetwork were decided as core genes with the strong capacity of modulating adjacent genes. Core genes in three networks shown are in Figure 4: 1) the left subnetwork, which has a core status made up of 15 core genes with the k-core of 6; 2) the bottom middle subnetwork; and 3) the right subnetwork, which has a core status made up of 10 core genes with the k-core of 9. After referring to the European Bioinformatics Institute (EBI) database, the majority of these 25 genes are involved in immune response (including hematopoiesis), response to stimuli and gene expression, which is consistent with the results of GO analysis (see details in Table 2).

**Table 2:** The hub genes and their biological functions in the two main k-core subnetworks.

Gene symbol	Gene description	Degree*	k-core	GO term
Trafd1	TRAF type zinc finger domain containing 1	18	9	response to cytokine stimulus
H2-T22	histocompatibility 2, T region locus 22	16	9	immune response
				antigen processing and presentation
Serpina3f	serine (or cysteine) peptidase inhibitor, clade A, member 3F	16	9	response to cytokine stimulus
Oas2	2′–5′ oligoadenylate synthetase 2	14	9	immune response/RNA catabolic process
Tap1	transporter 1, ATP-binding cassette, sub- family B (MDR/TAP)	14	9	protection from natural killer cell mediated cytotoxicity
				transmembrane transport
H2-T10	histocompatibility 2, T region locus 10	13	9	immune response
				antigen processing and presentation
Dtx3l	deltex 3-like (Drosophila)	12	9	chromatin modification
				response to DNA damage stimulus
AI451557 (Nlrc5)	NLR family, CARD domain containing 5	11	9	regulation of NF-kappaB transcription factor activity
				innate immune response
LOC56628 (H2-K1)	“histocompatibility 2, K1, K region”	11	9	positive regulation of T cell mediated cytotoxicity
				immune response
				antigen processing and presentation
Oasl2	2′–5′ oligoadenylate synthetase-like 2	11	9	immune response
Hist1h2bm	“histone cluster 1, H2bm”	14	6	nucleosome assembly
Dnajb9	DnaJ (Hsp40) homolog, subfamily B, member 9	12	6	protein folding
Ccnl1	cyclin L1	11	6	regulation of transcription
Klf2	Kruppel-like factor 2 (lung)	11	6	positive regulation of transcription
				erythrocyte homeostasis
Plk2	polo-like kinase 2 (Drosophila)	11	6	protein amino acid phosphorylation
				positive regulation of I-kappaB kinase/NF- kappaB cascade
Junb	Jun-B oncogene	10	6	osteoclast differentiation
				regulation of cell cycle
				response to cytokine stimulus
Hist1h4m	histone cluster 1, H4m	9	6	nucleosome assembly
Rhob	ras homolog gene family, member B	9	6	small GTPase mediated signal transduction
				cell differentiation
Hist1h4f	histone cluster 1, H4f	8	6	nucleosome assembly
Hist1h4k	histone cluster 1, H4k	8	6	nucleosome assembly
Hist1h1a	histone cluster 1, H1a	7	6	nucleosome assembly
Hist4h4	histone cluster 4, H4	7	6	nucleosome assembly
Egr1	early growth response 1	6	6	positive regulation of transcription from RNA polymerase II promoter
				T cell differentiation
Egr2	early growth response 2	6	6	regulation of transcription
Eif2ak2	eukaryotic translation initiation factor 2-alpha kinase 2	6	6	negative regulation of osteoblast proliferation
				protein amino acid autophosphorylation

*Degree: defined as the link numbers one node has to the other.

### An integrated network of irradiation-related genes

Radiation-related genes were extracted through PubMed database queries using four keywords: irradiation, ionizing radiation, gamma radiation and radiotherapy [26]. As shown in Table S9, there are 514 radiation-related genes of the species of mouse, rat, and human. The total protein-protein interaction (PPI) network of text-mined genes was constructed based on the Human Protein Reference Database (HPRD) to depict their complex relationship (Figure S1 and Table S10). A comparison between our experimentally derived differentially expressed genes and text-mined genes was conducted. As shown in Table S11, there are 42 differentially expressed genes in our microarray data set that have been confirmed to response to IR in published research. These genes are defined as overlapping genes. Eleven nodes in the gene co-expression network were included in this overlapping gene set, indicated in black outline (Figure 4). Interestingly, five genes—*Rhob*, *Egr1*, *Jun*, *Klf4* and *Fos* were clustered together.

**Figure 5:**
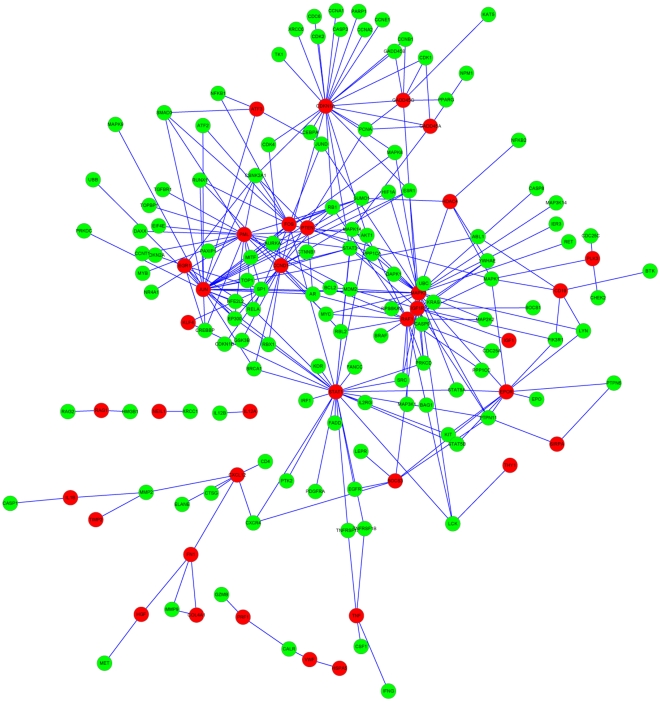
The PPI subnetwork extracted from the total PPI network of all the text-mined genes. The 42 overlapping genes were used as seed genes to construct a PPI subnetwork, which includes all the text-mined gene interactions with seed genes. Red nodes represent the overlapping genes; green nodes indicate gene interactions with overlapping genes that do not appear in the list of significant differential genes. Edges between them represent the protein-protein interaction. All information is based on HPRD.

We utilized these 42 overlapping genes as seed genes to extract a subnetwork (Figure 5) from the total PPI network (Figure S1) [27]. It is expected that these genes and their PPI networks will reliably associate with irradiation stimulus. Ten genes with degree values >10 based on the subnetwork are listed in Table 3 together with their GO terms.

**Table 3:** The key genes of the PPI subnetwork with degree >10 and their partial relative GO terms.

Gene symbol	Gene description	GO terms*
Jun	Jun oncogene	SMAD protein signal transduction/angiogenesis
		Regulation of proliferation and differentiation
Mapk3	mitogen-activated protein kinase 3	Cell cycle/intracellular signal transduction
Stat1	signal transducer and activator of transcription 1	JAK-STAT cascade
		cytokine-mediated signaling pathway
Cdkn1a (P21)	cyclin-dependent kinase inhibitor 1A (P21)	DNA damage response, signal transduction by p53 class mediator resulting in cell cycle arrest
		Negative regulation of apoptosis/cell proliferation
Pml	promyelocytic leukemia	induction of apoptosis/
		DNA damage response
		myeloid cell differentiation
Raf1	v-raf-leukemia viral oncogene 1	MAPKKK cascade/apoptosis
Ccnd1	cyclin D1	cell cycle/positive regulation of cell proliferation
Fos	FBJ osteosarcoma oncogene	cellular response to extracellular stimulus
		TGF beta receptor signaling pathway
Igf1r	insulin-like growth factor I receptor	immune response
		negative regulation of protein kinase B signaling cascade
Epor	erythropoietin receptor	erythropoietin receptor

*GO terms are based on PubMed database.

### Pathway analysis of 42 overlapping genes

After filtering out 42 overlapping genes by comparing significant differential genes and text-mined genes, we employed DAVID software on the basis of the KEGG pathway map to further investigate key pathways linked to these genes. Our analysis yielded 19 pathways, including cell adhesion and several pathways associated with immune response (Table 4). The importance of immune response at the recovery phase is highlighted by the identification of several immune-related signaling pathways that could be considered to be important for recovery, such as B/T cell receptor signaling pathway, acute myeloid leukemia, hematopoietic cell lineage and natural killer cell mediated cytotoxicity.

**Table 4:** The summary of significant pathway associated with 42 overlapping genes.

Pathway name	*p*-value	FDR	Gene symbol
Focal adhesion	4.50E-08	4.77E-05	VWF, IGF1R, CCND1, COL4A1, JUN, MAPK3, RAF1, IGF1, HGF, PTEN, FN1
Toll-like receptor signaling pathway	1.18E-05	0.0125	FOS, TNF, JUN, MAPK3, IL12A, IL1B, STAT1
p53 signaling pathway	2.90E-05	0.0307	CDKN1A, CCND1, GADD45G, IGF1, PTEN, GADD45A
MAPK signaling pathway	4.19E-04	0.443	FOS, TNF, JUN, GADD45G, MAPK3, RAF1, IL1B, GADD45A
B cell receptor signaling pathway	8.28E-04	0.875	FOS, CD19, JUN, MAPK3, RAF1
Acute myeloid leukemia	0.00343	3.58	CCND1, MAPK3, PML, RAF1
T cell receptor signaling pathway	0.00348	3.63	FOS, TNF, JUN, MAPK3, RAF1
Chronic myeloid leukemia	0.00769	7.86	CDKN1A, CCND1, MAPK3, RAF1
Jak-STAT signaling pathway	0.00853	8.69	CCND1, SOCS3, IL12A, EPOR, STAT1
Cytokine-cytokine receptor interaction	0.00915	9.30	TNF, IL12A, IL1B, EPOR, HGF, CXCL12
Hematopoietic cell lineage	0.0101	10.2	TNF, CD19, IL1B, EPOR
ErbB signaling pathway	0.0111	11.2	CDKN1A, JUN, MAPK3, RAF1
Natural killer cell mediated cytotoxicity	0.0273	25.5	PRF1, TNF, MAPK3, RAF1
Cell cycle	0.0309	28.3	CDKN1A, CCND1, GADD45G, GADD45A
NOD-like receptor signaling pathway	0.0437	37.7	TNF, MAPK3, IL1B
Fc epsilon RI signaling pathway	0.0718	54.6	TNF, MAPK3, RAF1
ECM-receptor interaction	0.0734	55.4	VWF, COL4A1, FN1
Chemokine signaling pathway	0.0737	55.6	MAPK3, RAF1, STAT1, CXCL12
GnRH signaling pathway	0.0959	65.7	JUN, MAPK3, RAF1

Among these pathways, focal adhesion molecules may have a role in bone marrow restoration by providing a structural link between membrane receptors and the actin cytoskeleton, or serving as signaling molecules, which regulate cell proliferation/differentiation and gene expression [28]. Interestingly, almost all of the differentially expressed genes with the function of cell adhesion were repressed, reaching nadir on 3^rd^ or 11^th^ day after IR, such as *Sirpa* (11^th^), *Itgb2* (11^th^), *Olfm4* (11^th^), *Amica1* (11^th^), *Col5al* (21^st^), *Mybpc2* (3^rd^), *Itgam* (11^th^), *Lyve1* (11^th^), *Nrp1* (11^th^), *Sdc3* (3^rd^), *Fn1* (11^th^) and *CD33* (11^th^). The decreased expression of the genes and the enrichment of focal adhesion suggest that HSCs might detach from their niches over a relatively long period after IR, then develop into various mature blood cells and aid to restore damage caused by radiation damage [29].

### Validation by Real-time RT-PCR

**Figure 6:**
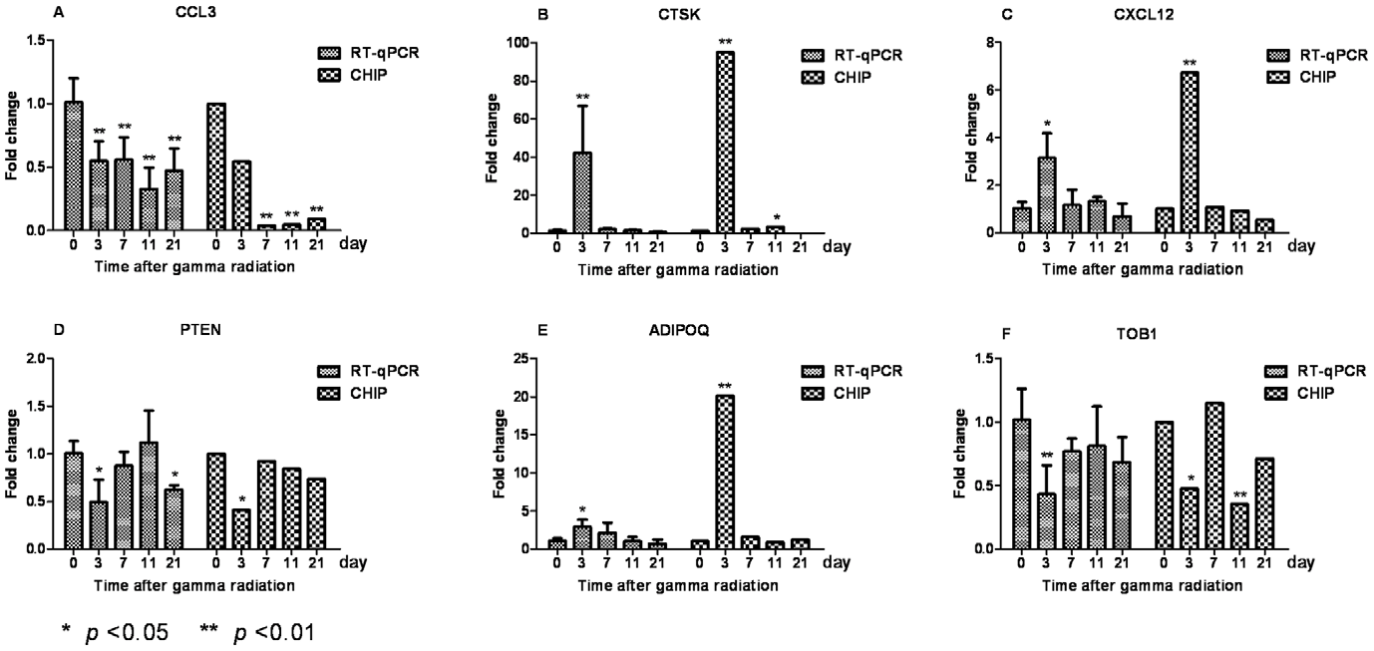
Validation of microarray results in BM after IR. A∼F show the expression fold changes of six differentially expressed genes: *Ccl3*, *Ctsk*, *Cxcl12*, *Pten*, *Adipoq* and *Tob1* at 3, 7, 11 and 21 DAI compared with sham-irradiated (0 DAI) samples (error bar represents one s.d.). The relative changes in gene expression were calculated by the 2^−ΔΔCt^ method, with *Gapdh* serving as an internal control gene.

To independently confirm the microarray results, real-time RT-PCR was performed on samples from BALB/c mice that had been exposed to the same experimental conditions that were used in microarray assay. The relative expression levels of six differentially expressed genes—*Ccl3*, *Ctsk*, *Cxcl12*, *Pten*, *Adipoq*, and *Tob1*—were assayed. Three genes—*Ctsk*, *Cxcl12*, *and Adipoq*—indicated significant early up-regulated changes at 3 DAI when compared to control (0 DAI) samples (*p*<0.05); and the other three genes—*Ccl3*, *Pten*, *and Tob1*—were significant down-regulated. It should be noted that the data were good consistent between the two experiments (Figure 6).

## Discussion

The high sensitivity of bone marrow to ionizing radiation can result in the eradication of hematopoietic cells, ultimately characterized by a severe shortage of white blood cells, platelets and red blood cells. Simultaneously, damaged bone marrow tissue can regenerate by activating HSC self-renewal, proliferation and differentiation pathways that depend upon cell-intrinsic and cell-extrinsic mechanisms [3], [30], [31]. According to cell-extrinsic mechanisms, HSCs are thought to reside within HSC niches, where supporting cells produce membrane-bound and secreted factors to regulate HSC biological processes (maintenance, differentiation and self-renewal) [31]. To comprehensively understand the response of BM to IR, especially in cells which constitute the hematopoietic niche, the whole bone marrow (mainly composed of bone marrow stromal cells) were subjected to IR in order to identify the differentially expressed gene sets. These genes are likely to play a central role in regulating the biological process of BM regeneration and promote organisms to recover from IR injury.

In our present experiments, we focused on the stage of BM regeneration, from the increase of the number of BMNCs at 3 DAI to 21 DAI, rather than irradiation-induced injury phase, which occurs within several hours after IR. Microarray analysis of irradiated mice at the recovery phase has not been report so far. Dai *et al.* reported that in murine BM 6 h after 6.5 Gy gamma irradiation, the functional categories of differentially expressed genes are comprised of biological processes including DNA replication/repair, proliferation/apoptosis, cell cycle control and RNA processing [8]. Therefore, we can conclude that the responses of irradiated bone marrow tissue are different at the stage of injury compared to recovery. For example, *p53* and its downstream target genes (such as, *cyclin G1* and *Bax*) which induce cell-cycle arrest, DNA repair and apoptosis are up-regulated after IR [8], [32], but we observed no expression change of these genes during the recovery phase in our experiment.

Microarray analysis is an effective technique to simultaneously compare the global gene expression between experimental and control groups. However, high-throughput data are generally of high variability, low reproducibility and contain non-specific noise [33], where subgroups of genes within the dataset may be associated with a generalized response to a given stimulus. This effect can be ameliorated using a comprehensive text mining and bioinformatics approach including: STEM cluster analysis, GO analysis, Dynamic Gene Network analysis and DAVID pathway analysis as an effective method to enrich the most relevant IR responsive genes.

STEM cluster analysis was performed for clustering and comparing the 1302 differentially expressed genes to depict the dynamic changes in these genes and their relationships to irradiation stimulus. Genes in the same temporal expression profiles are more likely to be grouped in certain pathways, or have common biological processes [34]. As shown in Table S3, we found that genes contained in the same temporal expression pattern share similar functionality. For example, profile 0 comprises 64 differentially expressed genes, with GO enrichment in immune response (including hemopoiesis) and cellular component organization.

Integrated results of GO analysis and cluster analysis suggest that immune response (including hematopoiesis), response to stimuli, cellular processes (cell proliferation, cell cycle, cell adhesion) and signaling processes are the most critical GO terms related to IR, especially for immune response at the recovery phase of IR. The conclusions were confirmed by GO map analysis, which is a method that can systemically construct the interaction network of the significant GO terms [35]. The high correlation between irradiation and immunity gene networks may result from the fact that HSCs are exceedingly sensitive to radiation. It is possible that IR exposure causes the depletion of lymphocytes and other immunocompetent cells within a short period, which in turn results in the depletion of immune responses [36]. In addition, activated immune cells have been reported to produce and secrete multiple hematopoietic cytokines, which have the ability to stimulate multilineage hematopoiesis cell recovery [37].

Dynamic Gene Network analysis is another method to demonstrate detailed interaction relationships among genes without depending on existing databases [38]. In our studies, there were 2 main k-core subnetworks obtained from our analysis, containing 25 key genes. Ten genes with the k-core of 9 are involved in immune response, and fifteen genes with the k-core of 6 (top in another subnetwork) regulate transcription and nucleosome assembly. According to the dynamic gene network, we can establish novel links between genes, which have never been reported. For example, *Fos* and *Ccl3* are connected together as shown in Figure 4, and we can find 7 papers in PubMed database by using keywords of “Fos” AND “Ccl3”. Therefore, it is probable that many novel genes or protein-protein interaction relationships related to IR exist.

Although research data related with irradiation have been accumulated dramatically during the past several decades, there were no literatures or databases with the integration of irradiation-related genes and proteins yet available. In addition, dynamic gene network was constructed by algorithmic prediction based on the gene expression profiles, which leads to the flexibility of the network model [38]. Therefore, we integrated high-throughput molecular profiling, protein-protein interaction and pathway databases and text mining to determine the biological interaction between genes. On the basis of the gene list extracted from literature, there are 514 genes of the species of mouse, rat and human related to irradiation responses, eleven of which appeared in the dynamic gene network. Interestingly, five genes: *Egr1*, *Rhob*, *Jun*, *Klf4* and *Fos* assembled in the subnetwork with the k-core of 6 and have defined protein-protein interactions. For example, Kruppel-like factor 4 (*Klf4*) is a transcriptional factor, which plays a role in the important processes of cell differentiation, cell proliferation and stem cell maintenance, and is involved in reprogramming different kinds of differentiated cells into induced pluripotent stem (iPS) cells [39], [40]. Wong *et al.* reported that *Klf4* in stem cells maintain long-term proliferation potential by activating telomerase at the cost of repressing cell differentiation [41]. Therefore, our results might demonstrate that down-expression of *Klf4* constantly at the recovery phase leads to differentiation of stem cells and recovery of blood cells. Due to the great importance of immune process, other core genes are also worthy to be explored further. As an example, *Junb*, which normally limits hematopoietic stem cell proliferation, was down-regulated in our results [42]. We might presume that the expression of *Junb* was repressed after IR for the purpose of increasing the proliferation of long-term repopulating HSCs.

A comparison of genes filtered using text mining and differentially expressed genes obtained by microarray, identified 42 overlapping genes that were selected as key genes. Interestingly, nearly all of them belong to the function category of immune response (including hematopoiesis). This result further suggests that immune response can be considered as a critical biological process in bone marrow recovery. The physiological functions of the *Gadd45* family of genes, as key players in response to multiple stresses (e.g. gamma-irradiation), are mediated by interactions with partner proteins, including PCNA, CDK1, p21, MEKK4, and p38 [43]. Among these partner genes, only *p21* was selected in our microarray assay. *Gadd45* genes mainly affect the response of myeloid cells to various acute stimuli, and contribute to the survival of myeloid cells in response to genotoxic stress [43], [44]. However, the functional role of *Gadd45* in the stage of BM recovery has not been investigated.

The PPI subnetwork (Figure 5) was extracted from the total protein-protein interaction network by using 42 overlapping genes as seed genes, which reduced the complexity of the total network. The DAVID pathway analysis of 42 overlapping genes provided several essential irradiation-related pathways including focal adhesion and several immune-related signaling pathways. These results suggest that these pathways are important at the recovery of irradiation damage. For instance, focal adhesion plays an important role in regulating HSC homing, hematopoietic cells location in hematopoietic niche and regulation of cell motility, proliferation, differentiation and survival [28], [45].

Taken together, we demonstrated gene expression profiles of irradiated mice at the recovery phase by microarray assay, integrated with a multi-step bioinformatics strategy to understand irradiation-related hub genes, pathways and biological processes. Our results indicate that the gene expression profiles of irradiated bone marrow tissue are different at the stage of injury and recovery. Immune response was determined to play critical role in bone marrow recovery. Several hub genes were identified using different constraints. These genes are likely to have essential roles in corresponding pathophysiological processes, including HSC self-renewal, immune response and cell proliferation. The total protein-protein interaction networks provide basic information for genetic association studies performed using irradiation, which could act as an initial step for better deciphering the molecular mechanisms of irradiation response together with our microarray results.

## Materials and Methods

### Animals and Irradiation experiments

Ethics Statement: All animal studies complied with current ethical considerations with the approval (SYXK-2007-0025) of Shanghai Jiao Tong University School of Pharmacy. Male specific pathogen free (SPF) BALB/c mice, 7 to 8 weeks of age with body weights of 18∼22 g, were purchased from Sino-British SIPPR/BK Lab. Animal Ltd., Co. We used male mice to avoid the effect of an estradiol cycle. All mice had been bred under isolated conditions and housed in filter-top, micro-isolator cages.

Mice were randomly allocated to irradiated groups (n = 29 mice) and sham-irradiated (n = 6 mice) group as control reference. Mice were exposed to total body irradiation with a single dose of 4 Gy gamma-radiation or sham-irradiation (0 Gy). Irradiation treatment was performed at The Second Military Medical University by using a ^60^Co source with a dose rate of 0.7653 Gy/min. At 3, 7, 11 and 21 days after irradiation (DAI) or 0 day (sham-irradiation), 0.5–1 ml of blood was collected and the animals were euthanized. Whole bone marrow cells from both tibias were collected using a previously published protocol [8], [46]. Six to eight mice were used for each time point to collect enough cells for RNA extraction. Experiments were performed in accordance with the National Experimentation Manual.

### Blood cell counts

The regeneration of BM post-injury was assessed by means of peripheral blood cell counts and bone marrow nucleated cell (BMNC) counts. Peripheral blood samples were analyzed by using Automated hematology analyzer Celltac α (Nihon Kohden, Japan) according to the manufacturer's protocol. Bone marrow nucleated cells were calculated by using blood cell count board.

### RNA isolation

Bone marrow cells were added to TRIzol Reagent (Invitrogen, Carlsbad, CA, USA) in amount of 1∼3×10^7^ cells/ml of TRIzol. Total RNA was extracted according to the manufacturer's protocol, and was further purified by using RNeasy mini-columns (QIAGEN, Valencia, CA). The absorbance ratio at 260/280 was measured to evaluate the purity of all the RNA samples, and the quality and integrity were assessed by agarose gel electrophoresis. All the RNA samples exhibited an intensity ratio of 28S to 18S rRNA bands that was approximately 2∶1, and the OD260/OD280 ratio fell in the range of 2.0–2.1.

### Microarray experiment

RNA samples from four irradiated mice and one sham-irradiated mouse were used to detect gene expression changes at each time point. Gene expression profiling was performed using Illunima's MouseWG-6 Beadchips (Illumina Inc., San Diego, CA). The biotinylated and amplified cRNA was generated from the total RNA samples using the Illumina TotalPrep RNA Amplification Kit (Ambion, applied Biosystems, Foster City, CA). All the procedures were based on the manufacturer's RNA amplification protocol, consisting of reverse transcription to synthesize first strand cDNA (dsDNA) with a T7 Oligo(dT) Primer. The cDNA then underwent second strand synthesis and purification, followed by *in vitro* transcription with T7 RNA Polymerase to generate multiple copies of biotinylated cRNA.

After sample labeling, five labeled and amplified cRNAs samples were hybridized to Illumina Mouse-6 Expression Bead Chips, following the manufacturer's protocols. Images were extracted using the Illumina Iscan Reader and the final text file was output through Illumina's Genome Studio application after data normalization based on average normalization algorithms. The output file contained the original signal of each gene, including Average Signal, Detection P value, DiffScore and Symbol. Detective p-value was applied to describe the significant difference between signal intensity and background. Statistical significance was calculated using the Student's t test (*p*-value<0.05 for at least one time point), and *p*-value was corrected by false discovery rate (FDR). Microarray data were deposited in the GEO public database (accession number: GSE28195). All data generated in this study were MIAME compliant.

### Bioinformatics analysis of microarray data

**Figure 7:**
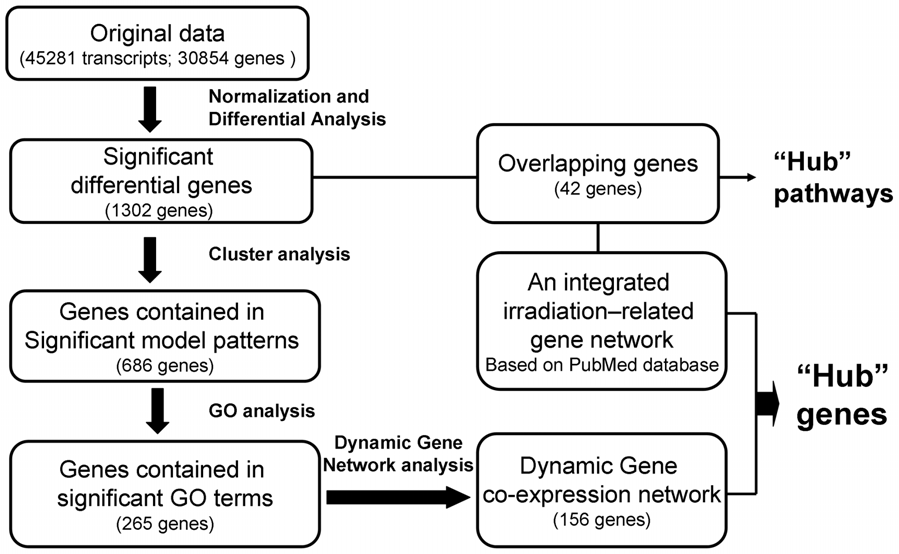
Flow chart of bioinformatics analyses for selecting hub genes and pathways related to IR.

A comprehensive bioinformatics analysis approach was used to enrich the dataset for genes that were most likely to be associated with recovery from IR, including: clustering analysis, Gene ontology (GO) analysis, GO map analysis and Dynamic Gene network analysis (Figure 7).

#### Cluster analysis

We subjected 1302 significant differential genes to cluster analysis using the Short Time-series Expression Miner (STEM) version 1.3.6 (http://www.cs.cmu.edu/~jernst/stem/). Fifty model temporal expression patterns were used to identify the expression patterns of significant differential genes. These were simultaneously integrated with Gene Ontology classification. Each cluster contained certain number of genes that have similar expression patterns after IR. The gene clusters were ranked by the *p*-value significance of the observed number of genes that fit a profile beyond the expected number.

#### GO analysis

A total of 686 genes, fell in significant model profiles (*p*<0.001), underwent Gene Ontology analysis according to the Gene Ontology, which is the key functional classification of NCBI [22]. All the GO terms assigned to these genes were obtained, and examined simultaneously by Fisher's exact test and 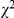 test for calculating the level of significance. The FDR was calculated to correct the *p*-value [47], the smaller the FDR, the smaller the error in judging the *p*-value. In addition, enrichment also provides a measure of the significance of the function: as the enrichment increases, the corresponding function is more specific under our experimental condition [35]. The criterion of *p*-value<0.05 was used to screen out significant GO terms. The manual summarization was conducted based on European Bioinformatics Institute (EBI) database (http://www.ebi.ac.uk/QuickGO/).

#### Dynamic gene network analysis

The normalized signal intensity of significant differential genes was used to build a co-expression network. At first, the Pearson's correlation of each pair of genes was calculated as the basis of choosing the significant correlation gene pairs. Then the gene-gene interaction network was established according to the correlation between genes. Within the network analysis, nodes represent the genes, and the edges between genes depict the interaction between them [48]. All the nodes were marked with degree, which is defined as the link numbers one node has to the other. Genes with higher degrees occupied more central positions in the network and had a stronger capacity of modulating adjacent genes. In addition, k-core in graph theory was applied to describe the characteristics of the network including, but not limited to, the centrality of genes within a network and the complexity of the subnetworks. According to the relationship between genes, they were divided into several subnetworks, and marked with different colors [25].

#### Pathway analysis

Overlapping genes were identified by a comparison between significant differentially expressed genes and text-mined genes. The pathways were enriched using the Database for Annotation, Visualization and Integrated Discovery (DAVID) v6.7 web server [49] (http://david.abcc.ncifcrf.gov/home.jsp), interrogating KEGG database.

### Establishment of an integrated gene network

The integrated gene list, which includes genes related to irradiation, was constructed by text mining. Four keywords of “irradiation”, “ionizing radiation”, “gamma radiation” and “radiotherapy” were used as search terms for collecting all the radiation-related literatures from PubMed database [26]. The total protein-protein interaction network of all the radiation-related genes was also constructed based on HPRD (http://www.hprd.org/), after mapping all the selected genes to human genes. We also constructed the subnetwork of 42 overlapping genes, and listed 10 core genes with the degree values >10 and their GO terms based on the PubMed database.

### Validation of microarray data by Real-time PCR

Real-time RT-PCR was performed to verify the differential expression of 6 selected genes, those are *Ccl3*, *Ctsk*, *Cxcl12*, *Pten*, *Adipoq* and *Tob1*. Total RNA was isolated from BM of BALB/c mice exposed to the same experimental conditions as used in microarray assay including three independent experiments. RNA was reverse transcribed using ReverTra Ace qPCR RT Kit (TOYOBO, Osaka, Japan). Real-time RT-PCR was conducted in 10 µl reactions consisted of 5 µl 2×SYBR Green Real-time PCR Master Mix (Applied Biosystems), 0.3 µM primers, and 1 µl template cDNA. The PCR cycling programs began with an initial denaturation for 5 min at 94°C, followed by 40 cycles of 30 sec at 94°C, 30 sec at 60∼65°C (listed in Table 5), 30 sec at 72°C, and ended with the step of melting curve. The relative changes in gene expression were calculated by 2^−ΔΔCt^ method, GAPDH was used as an internal control gene to normalize the amount of RNA added to the PCR reactions [50].

**Table 5:** Primer sequences.

Gene symbol	Direction	Primer sequence (5′-3′)	Product size (bp)
Ccl3	Sense	CAAGTCTTCTCAGCGCCAT	158
	Anti-sense	ATCTGCCGGTTTCTCTTAGTCA	
Ctsk	Sense	GGGCCAGGATGAAAGTTGTA	106
	Anti-sense	CACTGCTCTCTTCAGGGCTT	
Cxcl12	Sense	ACATCGCCAGAGCCAACGTCA	111
	Anti-sense	TCGGGTCAATGCACACTTGTCT	
Pten	Sense	ACTGCACGAATAATAAGGCAT	152
	Anti-sense	TAAAATTGAAGCCCTAATCCC	
Adipoq	Sense	CACTGGCAAGTTCTACTGCAA	140
	Anti-sense	TCTTTTCCTGATACTGGTCGT	
Tob1	Sense	TGCTCTTTCTCCCAATGCCAA	149
	Anti-sense	CTCCGTAGGCCGCAAACAC	
Gapdh	Sense	TGTGTCCGTCGTGGATCTGA	104
	Anti-sense	TTGCTGTTGAAGTCGCAGGAG	

## Supporting Information

Figure S1 **The protein-protein interaction network of text-mined genes related to IR.** The yellow nodes represent genes, and the blue line shows the interactions between genes. All information is based on HPRD.
(TIF)

Table S1 **Significant differential genes by comparison of irradiated samples and sham-irradiated samples.**
(XLS)

Table S2 **The analysis results of short time series gene expression data.**
(XLS)

Table S3 **The enrichment of Gene Ontology in each temporal expression pattern analyzed by STEM software.**
(XLS)

Table S4 **Significant functional categories and their differentially expressed genes according to biological process.**
(XLS)

Table S5 **The classification of functional categories.**
(XLS)

Table S6 **Significant functional categories (** ***p*** **<0.05) and their differentially expressed genes according to cellular component.**
(XLS)

Table S7 **The characteristics of GO map and the interaction among these significant GO terms.**
(XLS)

Table S8 **The genes contained in dynamic co-expression network and the functional categories of its genes with the degree >5.**
(XLS)

Table S9 **The irradiation-related genes extracted through text mining.**
(XLS)

Table S10 **The interaction between irradiation-related genes.**
(XLS)

Table S11 **Overlapping gene set obtained by comparing significant differential genes and text-mined genes.**
(XLS)
