# Supplementary figures and images for: Identification of Hub Genes Related to the Recovery Phase of Irradiation Injury by Microarray and Integrated Gene Network Analysis
Source: PLoS One. 2011 Sep 13;6(9):e24680. doi: 10.1371/journal.pone.0024680 (PMC3172286; doi:10.1371/journal.pone.0024680)

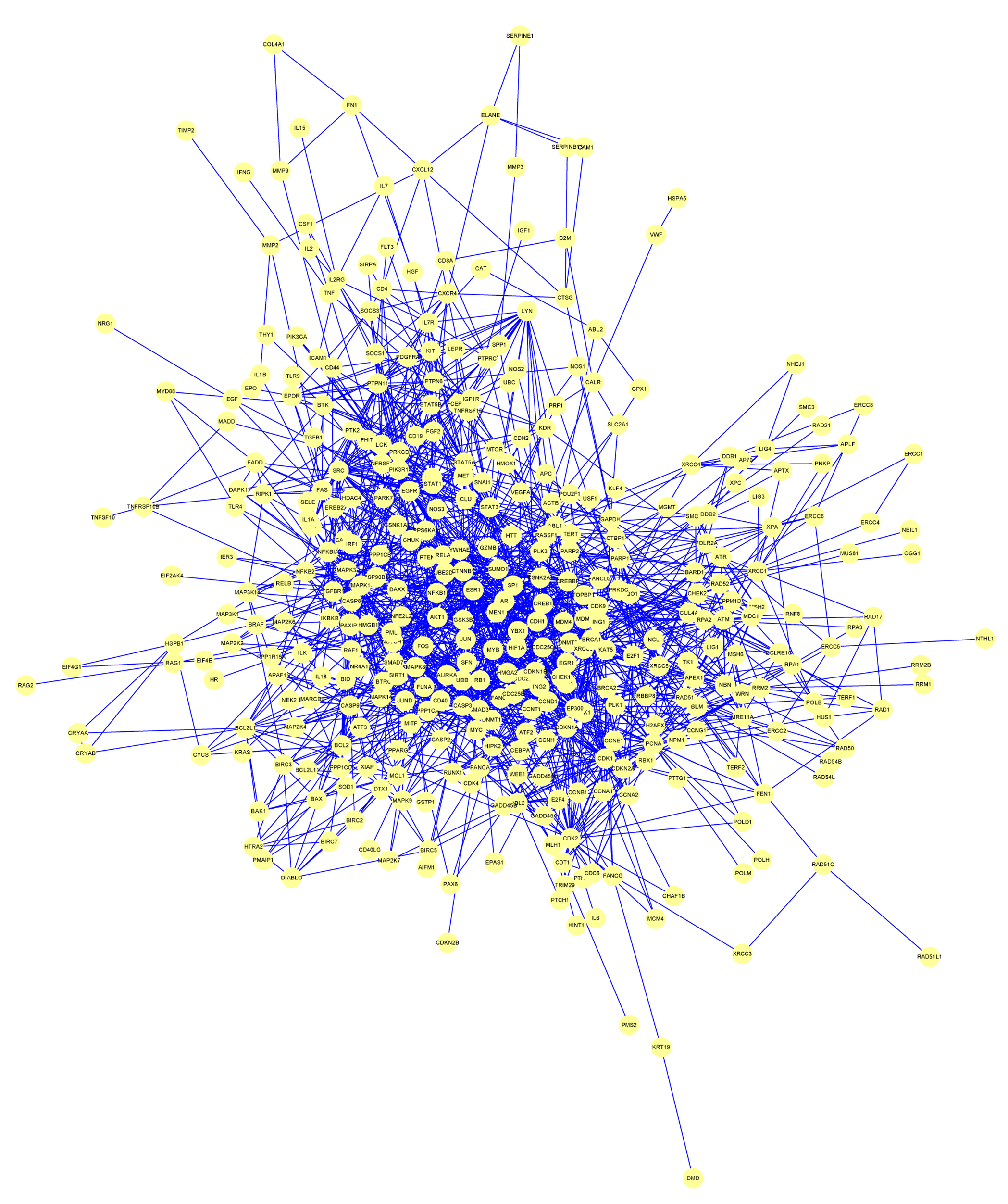

Supplement: Figure S1 — The protein-protein interaction network of text-mined genes related to IR. The yellow nodes represent genes, and the blue line shows the interactions between genes. All information is based on HPRD. (TIF) [file pone.0024680.s001.tif]
